# Prognostic value of endoglin-assessed microvessel density in cancer patients: a systematic review and meta-analysis

**DOI:** 10.18632/oncotarget.23546

**Published:** 2017-12-21

**Authors:** Jinguo Zhang, Lingyun Zhang, Qunbo Lin, Weimin Ren, Guoxiong Xu

**Affiliations:** ^1^Center Laboratory, Jinshan Hospital, Fudan University, Shanghai 201508, China; ^2^Department of Oncology, Shanghai Medical College, Fudan University, Shanghai 200032, China

**Keywords:** cancer, CD105, disease-free survival, overall survival, prognosis

## Abstract

**Background:**

Endoglin (ENG, CD105), an auxiliary receptor for several TGF-β superfamily ligands, is constitutively expressed in tumor microvessels. The prognostic value of ENG-assessed microvessel density (MVD) has not been systemically analyzed. This meta-analysis reviews and evaluates the association between ENG expression and prognosis in cancer patients.

**Materials and Methods:**

Thirty published studies involving in 3613 patients were included after searching of PubMed, Web of Science, and EMBASE. The pooled hazard ratios (HRs) and 95% confidence intervals (CIs) for overall survival (OS), disease-free survival (DFS), and cancer-specific survival (CSS) were calculated using random-effects models. The publication bias was detected by a Begg’s test and Egger’s test. The outcome stability was verified by sensitivity analysis.

**Results:**

The high ENG-assessed MVD was significantly associated with poor OS (HR = 2.14, 95% CI 1.62–2.81; *P* < 0.001), DFS (HR = 3.23, 95% CI 2.10–4.95; *P* < 0.001), CSS (HR = 3.33, 95% CI 1.32–8.37; *P* < 0.001). Furthermore, subgroup analysis revealed that the association between the overexpression of ENG in tumor microvessels and the outcome endpoints (OS or DFS) were also significant in the Asians and Caucasians patients with different cancer types.

**Conclusions:**

ENG of tumor microvessels is a predictor of poor OS, DFS and CSS and may be a prognostic marker of patients with cancer.

## INTRODUCTION

Cancer have become a major public problem worldwide and the second-largest killer in the United States of America with 1,688,780 new cancer cases and 600,920 new deaths in 2017 [1]. Angiogenesis, a hallmark of cancer, is a crucial step for tumor growth and plays an important role in cancer metastasis. Hence, blocking tumor angiogenesis is a strategy for cancer treatment. Many efforts have focused on finding specific angiogenic markers that can be exploited by vascular targeting drugs over the last few decades [2]. The counting of microvessel density (MVD) is one of the most representative methods to quantify angiogenesis in human cancer tissues [3]. This method requires vascular endothelium markers to visualize microvessels detected by immunohistochemistry (IHC). In addition, there is increasing evidences to show that MVD determination in human cancer specimens is significantly associated with patients outcome [4].

Endoglin (ENG), also known as CD105, HHT1 and ORW1, is a homodimeric transmembrane glycoprotein. The relationship between ENG and cancer has been shown [5, 6]. It is highly expressed in activated vascular endothelial cells but weakly or not at all expressed in normal quiescent vessels, and therefore, it has been suggested as an important angiogenesis marker [7]. Indeed, the recent studies indicate that targeting ENG suppresses tumor angiogenesis [8–10]. ENG is an auxiliary receptor of transforming growth factor-β (TGF-β) that binds to TGF-β1 and TGF-β3 [11]. It modulates TGF-β signaling by interacting with type I and type II TGF-β receptor. In human malignancies, ENG is expressed in intratumoral vessels and peritumor, which up-regulated by hypoxia and TGF-β stimulation [12]. These features have made it become a prime maker for prognosis, tumor imaging, and anti-angiogenesis therapy. Furthermore, many studies have found that ENG may act as a prognostic biomarker to predict patient outcome and has better value than traditional vascular markers, such as vascular endothelial growth factor (VEGF), CD31, and von Willebrand factor [13–15].

Most studies show that high MVD, determined by ENG, seems to correlate with a poor survival in patients with cancer [13–40]. However, others show a conflicting result [41, 42]. To date, the reliability and degree of the prognostic value of ENG-assessed MVD in human solid tumor have not been systemically analyzed. In view of the limited samples and discrete outcomes in individual studies, we performed a comprehensive meta-analysis to assess the prognostic value of elevated ENG-stained microvessels in human malignances.

## RESULTS

### Study selection and characteristics

**Figure 1:**
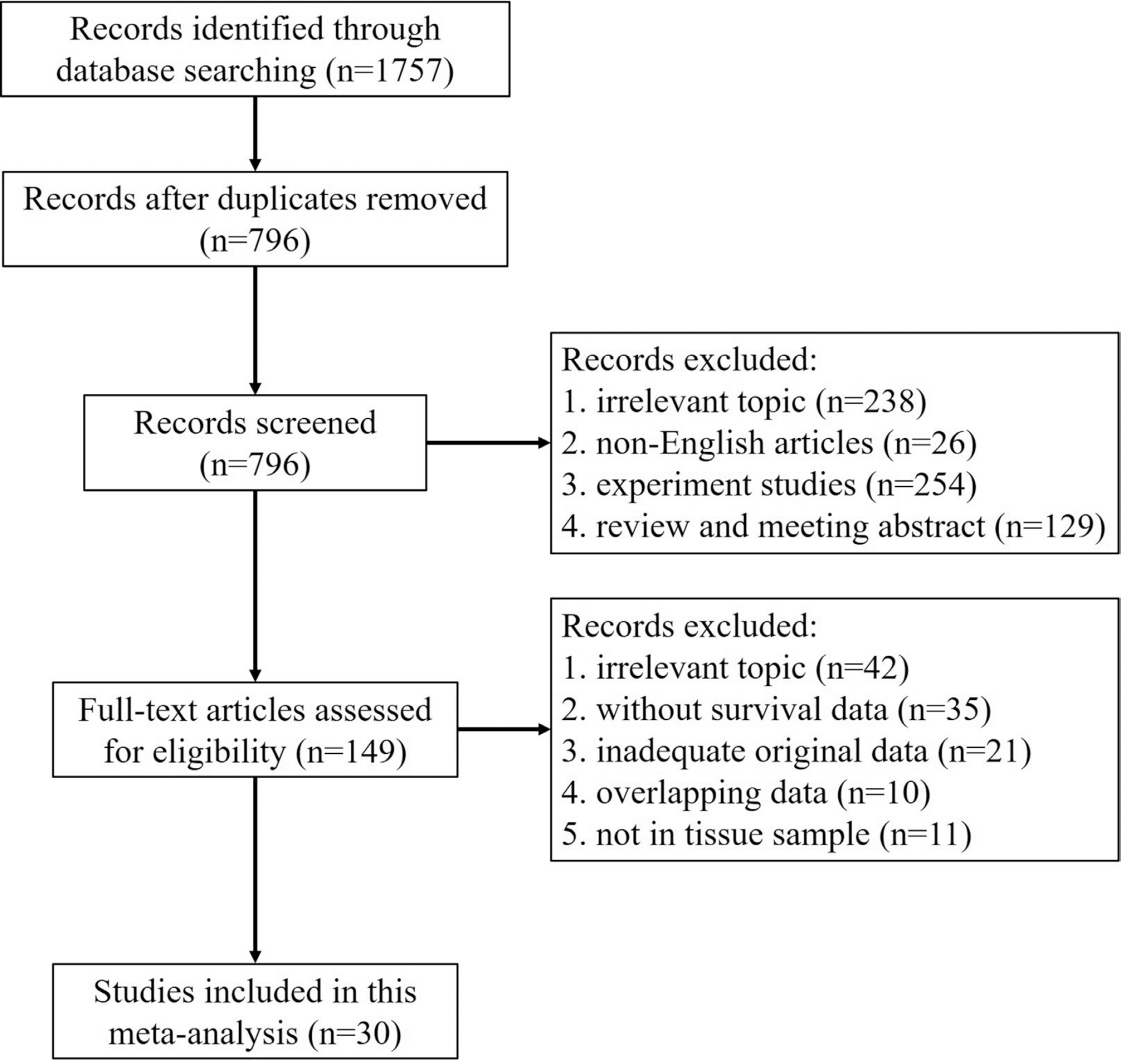
Flow diagram of the study selection procedure

Using the pre-defined search strategy, a total of 1757 articles were retrieved from PubMed, Web of Science, and EMBASE databases (Figure 1). After removed duplicate, screened and assessed for eligibility, a total of 30 studies encompassing 3613 patients with cancer were included in this meta-analysis. The median of sample size was 101 with a wide range from 36 to 929. All the included studies cover a wide range of race, region, and cancer type. Among all cohorts, European (53.33%) and Asian (36.66%) countries were the major source regions of literatures, followed by the USA (10.01%). We then evaluated these publications and found many types of cancers in the studies, including head and neck cancer (6 studies), esophagus cancer (3 studies), breast cancer (3 studies), colorectal cancer (3 studies), pancreatic cancer (2 studies), cervical cancer (2 studies), prostate cancer (2 studies), renal cancer (2 studies), gastric cancer (2 studies), ovarian cancer (1 study), urothelial cancer (1 study), glioblastoma (1 study), endometrial cancer (1 study), lung cancer (1 study). Among these studies, 21 studies had the data of OS, 11 studies had the data of DFS, and 3 studies had the data of CSS. The main characteristics of the included studies were shown in Table 1.

**Table 1:** Characteristics of studies included in the meta-analysis

Study	Year	Country	Case	Cancer type	Stage/Grade	Cut-off value	Follow-up time(range)	Multi Anal	S of HR	SO	NOS score
Behrem	2005	Croatia	46	GBT	NR	Mean	NR	no	SC	OS	6
Beketic	2011	Croatia	40	BC	G1-G3	ROC	55.8m (10.3–83.5)	no	R	OS	6
Chen	2014	China	124	EsCa	I-IV	Median	NR	no	SC	OS	6
Chuang	2006	Taiwan	94	TC	I-II	Mean	44.1m	yes	R	DFS	6
Dales	2003	France	929	BC	G1-G3	log-rank test	11.3y (6–15)	no	SC	OS/DFS	8
Dassoulas	2010	Greece	99	CRC	I-IV	NR	25.29m(1–63)	yes	SC	CSS	7
Erdem	2006	Turkey	90	EnC	I-IV	Quartiles	60.5m	yes	SC	OS	8
El-Gohary	2009	American	50	PCa	I-IV	Median	54.4m	no	SC	OS	7
Kyzas	2006	Greece	108	HNSCC	I-IV	Median	24m	yes	R	OS	7
Koyama	2010	Japan	132	GC	I-III	Median	65.23m (1–213)	yes	R	DFS	7
Li	2003	England	111	CRC	Dukes A-D	Median	60m	yes	SC	OS	8
Lin	2013	China	80	CC	I-IV	ROC	86m(2–144)	yes	R	OS	7
Lovato	2015	Italy	46	LC	pT1- pT4	ROC	66.9m	yes	R	DFS	6
Miyata	2013	Japan	122	UC	pT1- pT4	Mean	50m(2–250)	yes	R	CSS	7
Marioni	2010	Italy	108	LC	I-IV	ROC	38.0m	no	R	DFS	7
Martone	2005	Italy	127	HNSCC	I-IV	Median	70.8m (1–174)	yes	R	OS/DFS	8
Mineo	2004	Italy	51	NSCLC	I-II	Median	48.1m (4–150)	no	SC	OS	6
Martinovic	2015	Croatia	95	RC	II	ROC	54.7 ± 23.1m	yes	R	OS	7
Nikiteas	2007	Greece	100	GC	I-IV	Median	32.57 ± 29.57m	yes	R	OS	7
Randall	2009	American	173	CC	I-II	NR	NR	yes	R	OS	8
Rau	2012	Taiwan	140	BC	I-II	Median	NR	no	SC	OS	6
Saroufim	2014	France	102	ccRCC	I-IV	Tertiles	52m(4–90)	yes	R	OS/DFS	9
Saad	2005	American	75	EsCa	I-IV	Median	27.3 ± 10.2m	yes	SC	OS	7
Sakurai	2014	Japan	142	EsCa	I-IV	Mean	41m(1–137)	no	SC	OS/DFS	7
Taskiran	2006	Turkey	51	OC	I-IV	Quartiles	34m	yes	R	OS	7
Vayrynen	2016	Finland	148	CRC	I-IV	ROC	NR	no	R	DFS	9
Wikstro	2002	Sweden	72	PCa	I-IV	Median	NR	no	SC	CSS	8
Yoshitomi	2008	Japan	36	PanC	I-IV	Mean	NR	no	SC	OS/DFS	7
Zvrko	2009	Montenegro	80	LC	I-IV	Median	27m(6–60)	yes	R	DFS	7
Zhou	2015	China	42	PDAC	I-IV	Median	NR	no	SC	OS	6

Abbreviations: BC, breast cancer; CC, cervical cancer; ccRCC, clear-cell renal cell carcinomas; CRC, colorectal cancer; CSS, cancer-specific survival; DFS, disease-free survival; EnC, endometrial cancer; EsCa, esophageal cancer; GBT, glioblastoma; GC, gastric cancer; HNSCC, head and neck squamous cell carcinoma; HR, hazard ratio; LC, laryngeal carcinoma; m, month; Multi Anal, multivariate analysis; MVD, microvessel density; NOS, Newcastle-Ottawa-Scale; NR, not report; NSCLC, non-small cell lung cancer; OS, overall survival; OC, ovarian cancer; PanC, Pancreatic cancer; PDAC, pancreatic ductal adenocarcinoma; PCa, Prostate cancer; R, reported; RC, rectal cancer; ROC, receiver operating characteristic; SC, survival curve; SO, survival outcome; S of HR, source of HR; TC, tongue cancer; UC, urothelial cancer; y, year.

The quality of the 30 eligible studies was assessed by the NOS. The quality scores in total ranged from 6 to 9, indicating a highly methodological quality of included studies (Table 2).

**Table 2:** Methodological assessment by Newcastle-Ottawa scale

Study	Selection	Comparability	Outcome	Total
Behrem	2	2	2	6
Beketic	2	2	2	6
Chen	1	2	3	6
Chuang	2	2	2	6
Dales	3	2	3	8
Dassoulas	3	1	3	7
El-Gohary	2	2	3	7
Erdem	4	1	3	8
Huang	3	1	2	6
Koyama	4	1	2	7
Kyzas	2	2	3	7
Li	3	2	3	8
Lin	2	2	3	7
Lovato	3	1	2	6
Marioni	3	1	2	6
Martinovic	3	2	2	7
Martone	3	2	3	8
Mineo	2	1	3	6
Miyata	2	2	3	7
Nikiteas	2	2	3	7
Randall	3	2	3	8
Rau	3	2	1	6
Saad	3	2	2	7
Sakurai	2	2	3	7
Saroufim	3	2	3	8
Taskiran	3	2	2	7
Vayrynen	3	2	4	9
Yoshitomi	2	2	3	7
Zhou	2	2	2	6
Zvrko	3	2	2	7

Number indicates a quality score assessed from published articles.

### Association of ENG-assessed MVD on survival and heterogeneity

**Figure 2:**
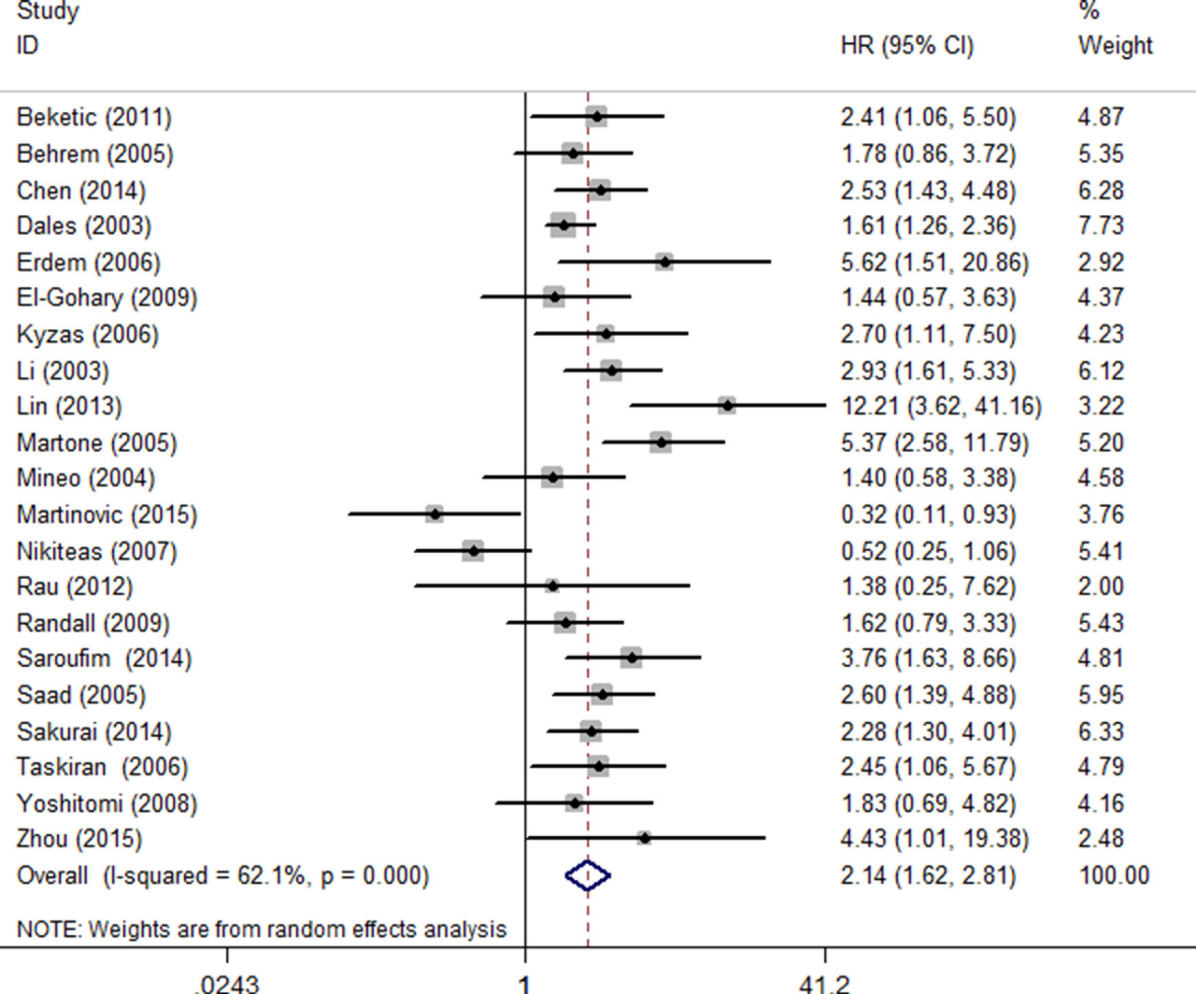
Forest plot of studies evaluating the pooled hazard ratios of high endoglin-MVD expression in solid cancers for OS Values of I^2^ and P and the HRs with their 95% CI of overall survival (OS) in various malignant tumors. A square represents a single study; the centre shows the HR with the horizontal lines denoting the 95% CIs. The diamond represents the overall HR for combined results of each study; the centre shows the HR and the extremities show the 95% CIs. HR, hazard ratio; CI, confidence interval.

The effect of high ENG-assessed MVD on OS was evaluated in 21 studies with 2712 patients. A random effects model was utilized to calculate the pooled HRs and 95% CIs because of the significant heterogeneity among studies (I^2^ = 62.1%, *P* = 0.000). The results showed that a high ENG-assessed MVD was associated with poor OS in cancer patients (HR = 2.14, 95% CI 1.62–2.81, *P* < 0.001) (Figure 2).

**Figure 3:**
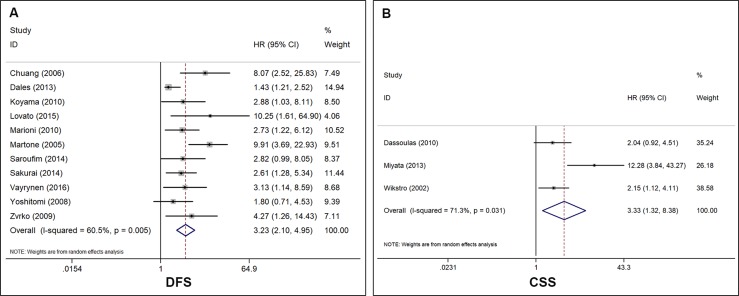
Forest plot of studies evaluating the hazard ratios of high endoglin-MVD in solid cancers for DFS and CSS (**A**) Values of I^2^ and P and the HRs with their 95% CI of disease-free survival (DFS). (**B**) Values of I^2^ and P and the HRs with their 95% CI of cancer-specific survival (CSS). A square represents a single study; the centre shows the HR with the horizontal lines denoting the 95% CIs. The diamond represents the overall HR for combined results of each study; the centre shows the HR and the extremities show the 95% CIs. HR, hazard ratio; CI, confidence interval.

The effect of high ENG-assessed MVD on DFS was evaluated in 11 studies with 1944 patients. A random effects model was applied to calculate the pooled HRs and 95% CIs because of the significant heterogeneity among studies (I^2^ = 60.5%, *P* = 0.005). The results indicated that the overexpression of ENG protein in tumor microvessels was significantly associated with poor DFS in patients with cancer (HR = 3.23, 95% CI 2.10–4.95, *P* < 0.001) (Figure 3A). The effect of high ENG-assessed MVD on CSS was evaluated in 3 studies with 293 patients. Because the heterogeneity test reported the *P* value of 0.031 and I^2^ value of 71.3%, a random effects model was applied to calculate the pooled HRs and 95% CIs. The pooled result showed that patients with high ENG-assessed MVD possessed a significantly shorter CSS compared with those patients with low ENG-assessed MVD (HR = 3.33, 95% CI 1.32–8.38, *P* < 0.001) (Figure 3B).

### Subgroup analyses of survival and heterogeneity

**Figure 4:**
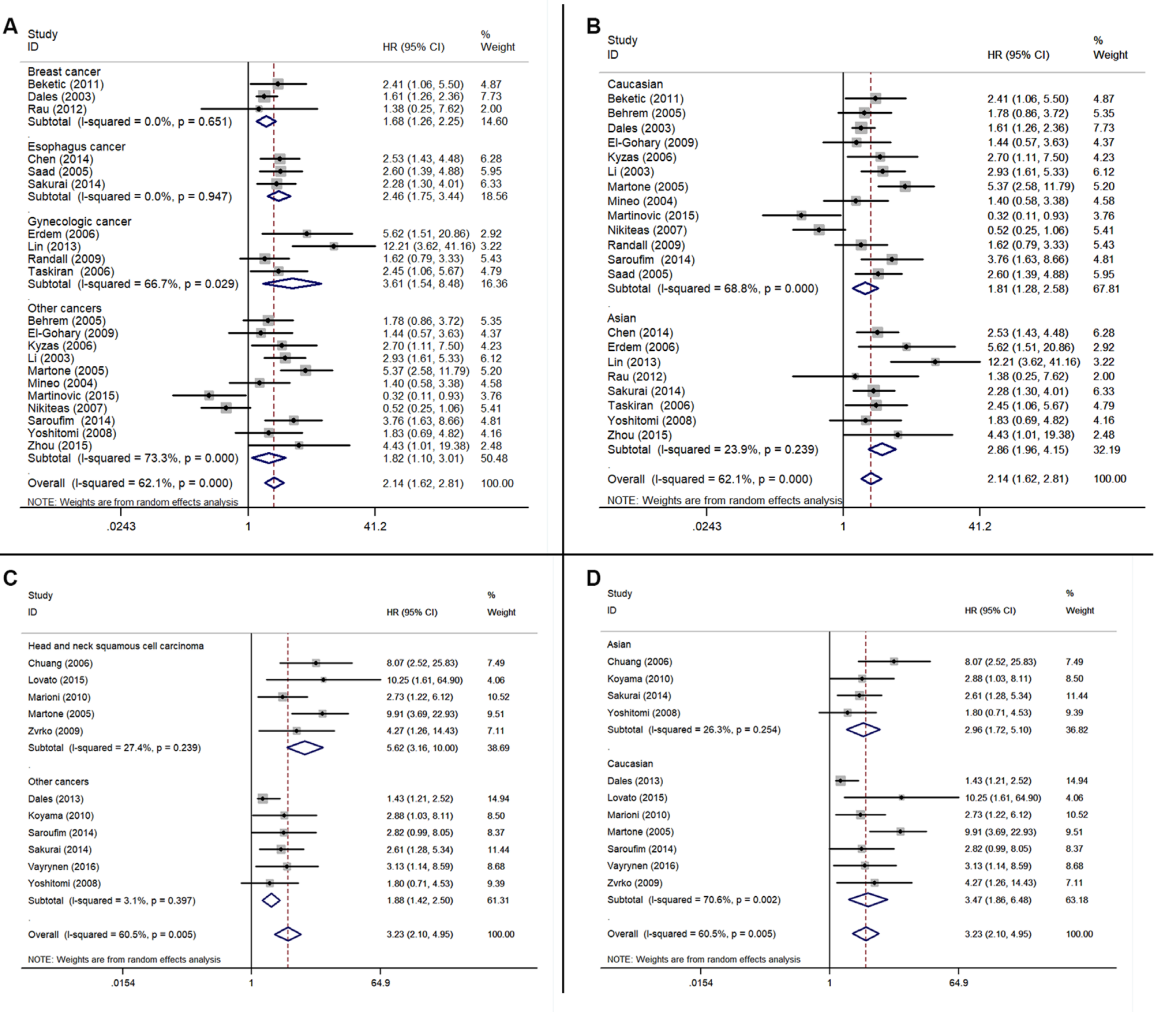
Forest plots showing the subgroup analyses of the relationship between the elevated endoglin-MVD expression and OS/DFS (**A**) Subgroup analysis of different cancer types for OS. (**B**) Subgroup analysis of the origin of patients for OS. (**C**) Subgroup analysis of different cancer types for DFS. (**D**) Subgroup analysis of the origin of patients for DFS. A square represents a single study; the centre shows the HR with the horizontal lines denoting the 95% CIs. The diamond represents the overall HR for combined results of subgroup study; the centre shows the HR and the extremities show the 95% CIs. HR, hazard ratio; CI, confidence interval.

In the subgroup analysis of survival by cancer types, we found that high ENG-assessed MVD was associated with poor OS of patients with breast cancer (HR = 1.68, 95% CI 1.26–2.25, *P* < 0.001), esophagus cancer (HR = 2.46, 95% CI 1.75–3.44, *P* < 0.001), gynecologic cancer (HR = 3.61, 95% CI 1.54–8.48, *P* = 0.003), other cancers (HR = 1.82, 95% CI 1.10–3.01, *P* = 0.019) (Figure 4A). Furthermore, in the subgroup analysis of survival by the origin of patients, we also found that high ENG-assessed MVD was associated with poor OS of Caucasian (HR = 1.81, 95% CI 1.28–2.58, *P* = 0.001) and Asian (HR = 2.86, 95% CI 1.96–4.15, *P* < 0.001) (Figure 4B). Similarly, in the subgroup analysis of DFS by cancer type, we found that an increased ENG expression in tumor microvessel was associated with poor DFS of patients with head and neck squamous cell carcinomas (HR = 5.62, 95% CI 3.16–10.00, *P* < 0.001) and other cancers (HR = 1.88, 95% CI 1.42–2.50, *P* < 0.001) (Figure 4C). In the subgroup analysis of DFS by the origin of patients, an increased ENG expression in tumor microvessel was associated with shorter DFS of Asian (HR = 2.96, 95% CI 1.72–5.10, *P* < 0.001) and Caucasian (HR = 3.47, 95% CI 1.86–6.48, *P* < 0.001) (Figure 4D).

To further explore the source of the heterogeneity of OS and DFS, a meta-regression analysis was performed with the covariates including publication year, the origin of people, the number of patients, and multivariate analysis. For OS and DFS, none of these covariates could explain a significant source of the heterogeneity, except a multivariate analyses that showed the heterogeneity on DFS (Coef = 0.939, *P* = 0.014) (Table 3).

**Table 3:** Meta-regression analysis of endoglin-assessed MVD in cancer patients

		OS			DFS	
	Coef	Std.Err	*P*	Coef	Std.Err	*P*
Publication year	0.437	0.284	0.141	–0.123	0.430	0.781
Country	–0.368	0.314	0.255	0.124	0.444	0.786
No. of patient	–0.019	0.327	0.953	–0.361	0.468	0.460
Multivariate	0.470	0.306	0.141	0.939	0.309	0.014

Abbreviations: Coef, coefficient; DFS, disease-free survival; No, number; OS, overall survival; P, a value of probability; Std.Err, standard error.

### Publication bias and sensitivity analysis

**Figure 5:**
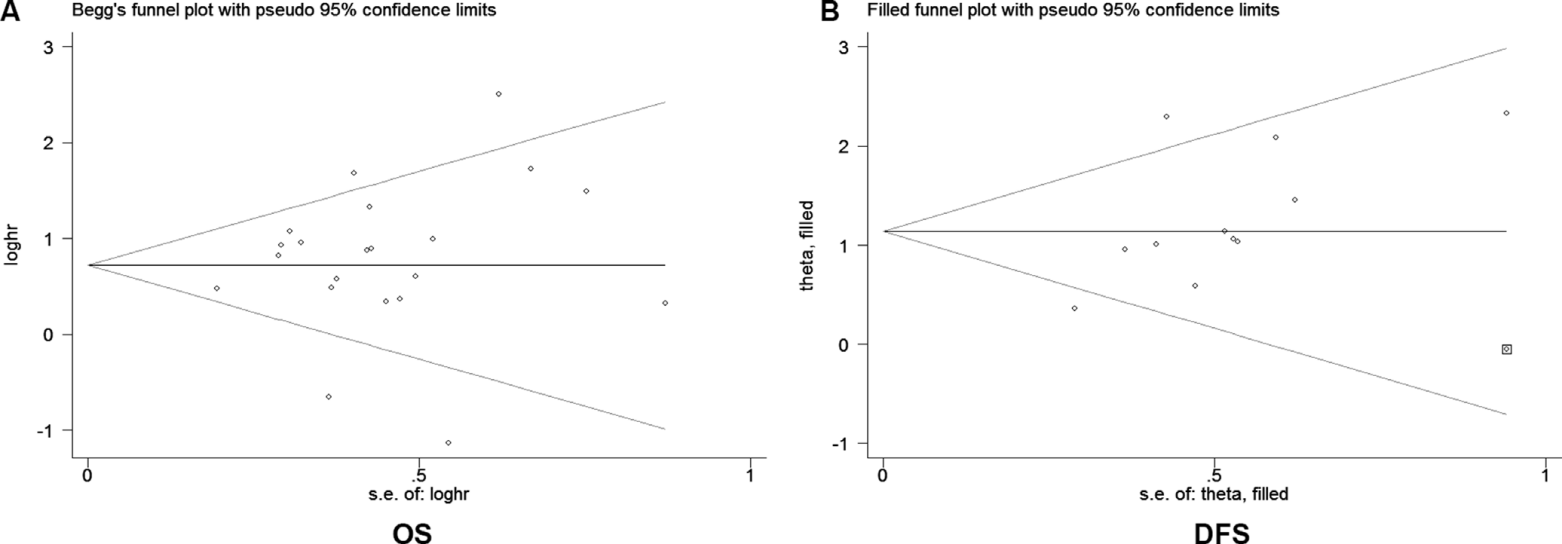
Begg’s funnel plot for publication biases (**A**) Funnel plot analysis for overall survival (OS). (**B**) Trim and filled method for disease-free survival (DFS). Circles represent the weight of the studies and square dots represent the added studies. Circles represent the identified studies and square dots represent the added studies after adjustment for publication bias. loghr, logarithm of hazard ratios; s.e., standard error.

The publication bias was evaluated by the Begg’s funnel plot and Egger’s test. The funnel plot for OS showed that there was no significant asymmetry (Figure 5A) and no significant publication bias was detected using Egger’s test (*P* = 0.486). However, the funnel plot for DFS showed slight heterogeneity (Figure 5B) and the results was validated by Egger’s test (*P* = 0.044). Therefore, the “Trim and Fill” method was used to adjust for publication bias. Without “deleted studies”, the pooled HRs remained stable.

**Figure 6:**
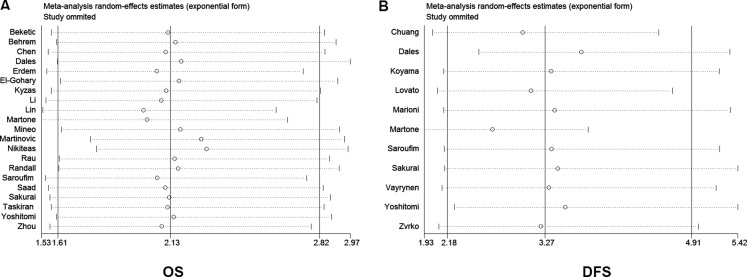
Sensitivity analysis of the meta-analysis Sensitivity analysis of the effect of the individual study influences on OS and DFS. (**A**) Sensitivity analysis of overall survival (OS). (**B**) Sensitivity of disease-free survival (DFS).

Next, we performed a sensitivity analysis to validate the robustness of study influences on OS and DFS. The removal of any individual study had no significant effect on the pooled results (Figure 6A and Figure 6B). These data indicated that no individual study was dominated by this meta-analysis.

## DISCUSSION

Current meta-analysis evaluates the prognostic value of ENG-assessed MVD in cancer patients. We collected 30 eligible studies with a total of 3613 patients and found that ENG-assessed MVD was significantly associated with OS, DFS, and CSS of patients with cancer.

Since the first study published in 1995 found the upregulation of ENG protein expression in tumor vascular endothelial cells [43], more and more studies have explored the role of ENG on the occurrence and development of tumors. Tumor invasion and metastasis, the most life-threatening events in tumorigenesis, are the major cause of the treatment failure in patients with cancer. Cancer cells shedding into the tumor vascular networks can initiate with the onset of tumor angiogenesis [44], which is permissive for the expansion of a tumor mass. For the anti-angiogenic therapies, much attention was focused on the VEGF family and the receptor tyrosine kinases [45]. In today’s anti-angiogenesis drugs, bevacizumab (a humanized monoclonal antibody directed against VEGF), sorafenib and sunitinib (two drugs targeting multiple receptor tyrosine kinases) have been applied to adjuvant chemotherapy with modest survival benefits [46]. In addition, the TGF-β signaling pathway is also involved in the differentiation of the endothelial cells and plays an important role in angiogenesis. Unlike these angiogenesis molecules, ENG binds to TGF-β1 and -β3, interacting with the signaling complex of TGF-β receptors types I and II [47]. Mice lacking ENG die from defective vascular development [48, 49]. In humans, hereditary haemorrhagic telangiectasia type 1 (HHT1), which is characterized by vascular malformations, is attributed to the mutation of the ENG gene [50]. Therefore, ENG-assessed MVD has been correlated with the angiogenesis of malignant tumor.

Unlike pan-endothelial marker, such as CD31, CD34, and von Willebrand factor, CD105 (ENG) is a marker of activated endothelium and participates in angiogenesis [7]. In the last two decades, some studies failed to find a correlation of high MVD with poor prognosis and even had a controversial issue on whether the high MVD in tumor is associated with poor prognosis [36, 51]. These were most likely due to the use of pan-endothelial markers which were inefficient in recognizing angiogenic endothelial cells. In current meta-analysis, the prognostic value of high ENG-assessed MVD for patients with breast cancer, esophagus cancer, gynecologic cancer, head and neck squamous cell carcinomas was dramatically remarkable. Apart from the prognostic value in cancer patients, ENG-assessed MVD is also positively associated with tumor stage, histopathological grade, and lymph node metastasis [52]. Recently, a chimeric monoclonal antibody (TRC105) targeting ENG has entered clinical trial to treat patients with advanced urothelial carcinoma [53], indicating that ENG may be a promising anti-angiogenic target of cancer therapy.

However, there are some limitations in our meta-analysis. First, the major limitation is the moderate heterogeneity of included studies. Using random effects model and meta-regression analysis, we explored the sources of heterogeneity. In view of the results of subgroup analysis, different cancer types and origin of patients contribute to the heterogeneity of OS and DFS. Second, this meta-analysis included 3613 patients of 30 studies, leading to the limited data in the subgroup analysis. Third, because several HRs were extracted by the survival curves rather than directly obtained from the primary studies, few statistical errors might be inevitable. Fourth, the cut-off values of ENG-assessed MVD were different. The median of MVD was most commonly used among the studies. Therefore, studies with larger-sample size are needed to determine the most suitable cut-off value. Finally, most of our included studies were retrospective studies, which are more likely to cause the publication bias. Thus, the association between the high ENG-assessed MVD and poor outcome might be overestimated.

In summary, this is the first meta-analysis with strong evidence that increased ENG protein expression in tumor microvessel is correlated to the poor OS, DFS, and CSS. ENG-assessed MVD is valuable prognostic indicator. Due to some limitations in small-sample sized studies, more high-quality, multiple-center, large-sample randomized, controlled trials should be conducted to confirm these results.

## MATERIALS AND METHODS

Current meta-analysis was done in agreement with the Systematic Reviews and Meta-Analyses (PRISMA) guidelines [54].

### Literature searching strategies

PubMed, Web of Science, and EMBASE databases were thoroughly searched from January 2000 to February 2017 using the search terms (endoglin or ENG or HHT1 or ORW1 or CD105) AND (cancer or tumor or tumour or carcinoma or malignant or malignancy or neoplasm) AND (prognostic or prognosis or survival) AND (mortality or outcome). We also identified potential studies through screening the reference list of identified articles. The comprehensive database search was performed independently by two individuals.

### Inclusion and exclusion criteria

The inclusion criteria were the following: (a) Evaluating of ENG-assessed MVD in tumor tissues for predicting patients prognosis; (b) ENG measurement by immunohistochemistry in tumor tissues; (c) Studies reporting survival data; (d) Studies reporting or containing sufficient data for extracting or calculating the HRs and 95% CIs; (e) Studies published in English.

The exclusion criteria were the following: (a) Meta-analysis, review, conference abstract, case reports, letters to the editor, and experimental studies without patient data; (b) Inadequate survival data for extracting HRs and 95% CIs; (c) Duplicate publications or overlapping database; (d) ENG measurement in blood; (e) Articles in non-English.

### Methodological assessment

The quality of a study was assessed by the Newcastle-Ottawa-Scale (NOS) for case-control studies. The NOS categorized into three dimensions including the selection of the study groups, the comparability of the groups, and outcome [55]. The score of the NOS ranged from 0 (lowest) to 9 (highest). The studies with 6 scores or more were identified as high quality studies. The assessment was performed independently by two authors and the final result was achieved by consensus.

### Data extraction

Baseline characteristics of 30 eligible studies was collected, including first author’s surname, publication year, origin country, case number, cancer type, detection methods, cut-off value of ENG overexpression, follow-up period, tumor stage, outcome, multivariate analysis or not, HRs and 95% CIs in the high ENG expression group versus the low ENG expression group. We analyzed three outcome endpoints: OS, DFS, and CSS. The cumulative recurrence was combined with DFS. The pooled HRs and 95% CIs were obtained directly from publications or from Kaplan-Meier curves with adequate survival data. If the study only presented Kaplan-Meier curves, we used the Engauge Digitizer V4.1 to obtain survival data and the Tierney’s methods [56] to calculate the HRs and 95% CIs. If the results from both univariate and multivariate analyses, we selected the result of multivariate analysis. Any disagreements between the two researchers were resolved by consensus review.

### Statistical analysis

The pooled HRs and 95% CIs for three outcome endpoints (OS, DFS, and CSS) were determined by random effects model. The heterogeneity across studies was tested by performing the Chi-square test and by I^2^ statistics [57, 58]. If the *P*-value was less than 0.01 and I^2^ value was more than 50%, the heterogeneity was considered to be significant. I^2^ values of < 25%, < 50%, and > 50% can also be considered as a low, moderate, and severe heterogeneity, respectively. The fixed effects model was performed according to the absence of the heterogeneity. Subgroup analysis and meta-regression were conducted to explore the source of a significant heterogeneity. Publication bias was detected by assessing the asymmetry of funnel plot. In addition, we also conducted Begg’s and Egger’s tests to quantify publication bias. The Duvaland Tweedie trim-and-fill method [59] was used to calibrate the effect when publication bias existed. The sensitivity analysis was also performed to validate the stability of pooled outcomes. All the statistical analyses were processed via STATA version 12.0 (Stata Corporation, College Station, TX, USA). A *P*-value of less than 0.05 indicated statistical significance for comparison.
